# Short Interpregnancy Interval Following a Multifetal Pregnancy: Maternal and Neonatal Outcomes

**DOI:** 10.3390/jcm12072576

**Published:** 2023-03-29

**Authors:** Ari Weiss, Hen Y. Sela, Sorina Grisaru-Granovsky, Misgav Rottenstreich

**Affiliations:** 1 Department of Obstetrics & Gynecology, Shaare Zedek Medical Center, Faculty of Medicine, The Hebrew University of Jerusalem, Jerusalem 91031, Israel; 2 Department of Nursing, Jerusalem College of Technology, Jerusalem 9116001, Israel

**Keywords:** twin pregnancies, interpregnancy interval, pregnancy outcome, preterm delivery

## Abstract

Objective: To evaluate the maternal and neonatal outcomes of women with short interpregnancy intervals (IPI < 6 months) following a multifetal pregnancy. Study design: A multicenter retrospective cohort study of women with an index multifetal delivery and a subsequent singleton gestation between 2005 and 2021. The obstetrical outcomes of pregnancies following short IPI (<6 months) were compared to those with an IPI of 18–48 months. Additional analyses were also conducted for the other IPI groups: 7–17 months, and longer than 49 months, while women with an IPI of 18–48 months served as the reference group. The primary outcome was preterm birth (<37 weeks) rate. Secondary outcomes were other adverse maternal and neonatal outcomes. Univariate and multiple logistic regression analyses were performed. Results: Overall, 2514 women had a primary multifetal delivery with a subsequent singleton gestation at our medical centers; 160 (6.4%) had a short IPI, and 1142 (45.4%) had an optimal IPI. Women with a singleton gestation following a short IPI were younger, with lower rates of previous cesarean and fertility treatments. Women in the short IPI group had significantly higher rates of preterm birth <37 weeks, anemia (Hb < 11 gr%) on admission to the delivery room, and placental abruption. Multivariable logistic regression analysis demonstrated that short IPI is associated with an increased risk for preterm birth (aOR 2.39, 95% CI 1.12–5.11, *p* = 0.03). Conclusion: Short IPI following a multifetal gestation is associated with an increased risk for preterm birth in subsequent singleton pregnancy.

## 1. Introduction

Maternal and neonatal adverse outcomes such as preterm birth (PTB), gestational hypertension, and gestational diabetes are more common in twin gestation, despite the advance in modern healthcare [1].

While factors that affect adverse outcomes in twin gestations have been studied, maternal and neonatal outcomes of subsequent pregnancies have been less explored. 

Inter-pregnancy interval (IPI), defined as the interval between birth to the subsequent pregnancy, is well-known as a significant risk factor for the following pregnancy outcome of both the mother and the neonate in various populations [2,3,4,5,6,7]. Previous studies evaluated the associations between different IPIs on maternal and neonatal outcomes, specifically in singleton pregnancies, while the information about the effect of IPI following twin pregnancies is scarce.

Mothers with twin pregnancies may be more prone to nutrient depletion problems [8,9]. Furthermore, a relatively large proportion of twin pregnancies are achieved through assisted reproduction and are in women of advanced maternal age. All of the above calls for balancing the pros and cons of short IPI against advanced age, etc. Considering all these factors, a population with previous twin pregnancies may be in greater danger of adverse obstetrical outcomes in a subsequent pregnancy and thus requires more in-depth research. 

The aim of the current study is to assess the association between short IPI following a twin delivery with PTB and other adverse obstetrical outcomes. 

## 2. Material and Methods

This was a multicenter retrospective cohort study conducted at two obstetrical centers. Our medical centers serve similar areas and have had approximately 20,000 deliveries per year throughout the study period. 

The study cohort included all women who had a singleton pregnancy following a twin delivery between 2005 and 2021. Women with miscarriages between deliveries and charts with missing data were excluded.

Deliveries were allocated into two groups: Singletons deliveries following a short IPI (<6 months) from twin gestation and those following an optimal IPI (18–48 months).

Additional analyses were also conducted for the other IPI groups: 7–17 months, and longer than 49 months, while women with optimal IPI (18–48 months) served as the reference group [4,10,11,12,13]. 

For this study, we used the definition of IPI as the following: the months that passed from the twins’ delivery and the last menstrual period of the next singleton pregnancy. [14].

The primary outcome of this study was PTB of less than 37 weeks. We also studied PTB < 34 weeks and <28 gestational weeks.

The following data were extracted from the computerized medical database: cesarean delivery, vacuum-assisted vaginal delivery, anemia upon admission to the delivery room, transfusion of blood products, admissions of mothers to the intensive care unit (ICU), prolonged hospitalization as defined below, chorioamnionitis, and puerperal fever, perinatal death, 5-min Apgar score < 7, small for gestational age (SGA), congenital malformations, neonatal asphyxia, meconium aspiration, jaundice, transient tachypnea of the newborn, brachial plexus injury, mechanical ventilation, convulsions, hypoglycemia, sepsis, encephalopathy, intracranial hemorrhage, and neonatal intensive care unit (NICU) admissions.

The composite adverse maternal outcome was defined as at least one of the following: Placental abruption, chorioamnionitis, puerperal fever retained placenta/placental fragments, postpartum hemorrhage, blood products transfusion, maternal ICU admissions, and prolonged hospitalization (hospitalization length ≥ 7 days after a cesarean or >5 days after vaginal delivery). 

Composite adverse neonatal outcome was defined as at least one of the following: perinatal death, SGA, 5-min Apgar score < 7, neonatal asphyxia, meconium aspiration, jaundice, congenital malformations, transient tachypnea of the newborn, brachial plexus injury, mechanical ventilation, convulsions, hypoglycemia, sepsis encephalopathy, and intracranial hemorrhage.

### Statistical Analysis

Continuous variables were described as a mean and standard deviation, while those without normal distribution were presented as a median and interquartile range. We used the Student’s *t*-test and the Mann–Whitney U test for the analysis of continuous variables with and without normal distribution, respectively. Categorical variables are described as proportions significance was assessed using the χ^2^ test and Fisher’s exact test as appropriate. For all tests, a two-sided *p*-value of less than 0.05 indicated statistical significance. 

To test the independent association between short IPI and the primary outcome (PTB < 37 weeks) and two of the secondary outcomes—composite adverse maternal and neonatal outcomes, three separate multivariable logistic regression models were applied. All variables found to be significantly associated with the risk of each outcome in univariate analysis (not presented) were included. Accordingly, the multivariate models for risk assessment for PTB < 37 weeks were adjusted for Previous PTB < 37 weeks, previous cesarean delivery (CD), anemia on admission to labor (Hb < 11 gr%), hypertensive disorders of pregnancy, and previous miscarriages > 3. The following variables were associated with the composite maternal outcome (secondary outcome): Gravidity, parity, previous CD, and fertility treatments (any treatment or medical procedure used primarily to address infertility). The following variables were associated with the composite neonatal outcome (secondary outcome): maternal age, anemia (Hb < 11 gr%) on admission to labor, previous CD, hypertensive disorders of pregnancy, and diabetes (pre-gestational and gestational).

Adjusted Odds Ratios (aOR) and 95% confidence intervals (CI) were calculated. These data were analyzed using SPSS software (version 25 statistical package; IBM, Armonk, NY, USA).

The study protocol was approved by the local institutional ethics committee and performed in accordance with the principles of the Declaration of Helsinki (IRB: 0196-20-SZMC). 

## 3. Results

**Figure 1:**
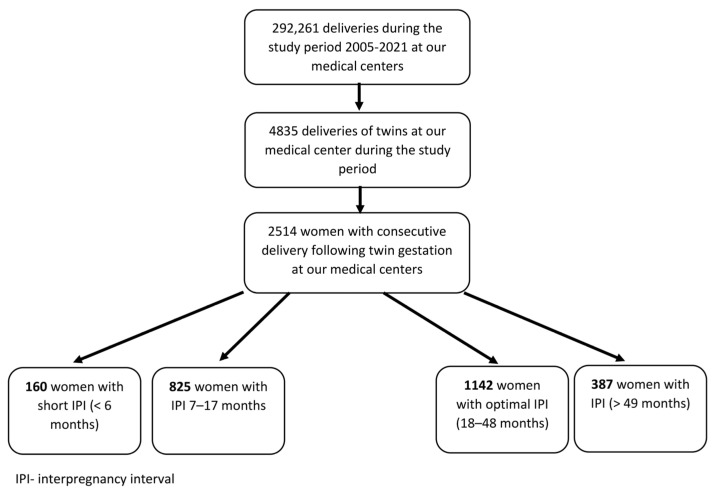
Flow chart of the study cohort.

During the study period, 2514 women had an index multifetal delivery followed by a singleton gestation in their next pregnancy and met inclusion and exclusion criteria (Figure 1); 160 women (6.4%) had a short IPI, and 1142 (45.4%) had an optimal IPI. 

Table 1 describes the characteristics of women, pregnancies, and deliveries in the cohort. Women in the short IPI group, compared to women with optimal IPI, were significantly younger, with a lower rate of conceiving following fertility treatments or previous CD and trial of labor after cesarean.

Table 2 shows the maternal outcomes of the women with singleton delivery following short versus optimal IPI from a twin delivery.

Women in the short IPI group had significantly higher rates of PTB < 37 weeks (9.7% vs. 4.3%, *p* < 0.01) as well as higher rates of indicated PTB (4.9% vs. 1.6%, *p* = 0.01), rates of PTB of less than 28, 32, 34 weeks or spontaneous PTB were comparable between the study groups. The rate of anemia (Hb < 11 gr%) on admission to delivery and placental abruption was also higher among women with short IPI. Composite adverse maternal outcomes rates were similar between the study groups.

All neonatal parameters examined did not differ between groups, including rates of composite adverse neonatal outcome (see Table 3).

Obstetrical characteristics and maternal and neonatal outcomes of the other IPI groups (7–17 months and longer than 49 months) versus women with optimal IPI are described in Supplementary tables (Tables S1–S3). 

Higher rates of anemia Hb < 11 gr% and Hb < 10 gr% on admission to delivery and lower rates of neonatal hypoglycemia were found among women with IPI of 7–17 months compared to women with optimal IPI. 

Women with IPI > 49 months had significantly higher rates of indicated PTB, both unplanned and elective CD, and significantly higher rates of NICU admission.

A multivariate model was calculated to examine the association between short IPI and PTB < 37 weeks, Table 4. The multivariate model confirmed that short IPI (<6 months) following twin pregnancies is independently associated with PTB < 37 weeks in the subsequent singleton pregnancy (2.39, 95% CI 1.12–5.11, *p* = 0.03).

Two additional multivariate models were used to assess the association between short IPI and composite adverse maternal and neonatal outcomes, Table 5. Short IPI following twin pregnancies was not found to be associated with either composite adverse maternal (1.47, 95% CI 0.89–2.42, *p* = 0.14) or neonatal outcome (1.19, 95% CI 0.70–2.04, *p* = 0.52).

## 4. Discussion

In this multicenter study, we evaluated the impact of short IPI following a twin delivery on the maternal and neonatal outcomes of the subsequent pregnancy and delivery. We found that a short IPI of less than six months is associated with an increased risk of PTB in <37 weeks in both the univariate and multivariate analysis compared to optimal IPI. In the univariate analysis, spontaneous and induced PTB were examined separately, with a significant association only to the induced PTB. In addition, women who conceived less than six months after a twin delivery had higher rates of anemia (Hb < 11 gr%) on admission to labor and placental abruption. Higher rates of anemia on admission to labor were also found in women with an IPI of 7–17 months in comparison to women with optimal IPI (18–48 months). We found no differences in the composite adverse maternal or neonatal outcomes between the different study groups.

Short IPI in singleton pregnancies has been shown repeatedly over the years to be associated with PTB, with most studies showing an association even after adjusting to potential confounders [5,11,15,16,17].

A recent study used a methodology of propensity score matching to isolate the contribution of IPI on PTB better and to determine the optimal IPI better. A dose-response relationship between short IPI and PTB was found, but IPI of more than 12 months was not associated with adverse outcomes, indicating that the optimal IPI might be shorter than studied so far [18]. 

The mechanism by which short IPI affects maternal and neonatal outcomes is yet to be determined. However, many theories exist. Conde-Agudelo et al. systematically reviewed these different outcomes and how they could be explained by each theory [19]. Conclusions of later studies were used to reinforce some of the theories.

For example, in a study that found a correlation between recurrent short IPI and PTB [20], it was argued that the findings of the study strengthened the “nutrient depletion theory,” and in a different study that found an association between short IPI and preterm premature rupture of membranes the conclusion was that the findings strengthened either the “cervical insufficiency theory” or a theory regarding the abnormal remodeling of the blood vessels in the endometrium [21].

Sufficient maternal nutrition during pregnancy, which is reflected via weight gain, is necessary for preventing adverse outcomes, including PTB [22]. It is plausible to assume that the nutrient depletion theory assumed to explain the PTB with singleton IPI may also explain the PTB with short IPI following twin delivery. As in twin pregnancies, the nutritional demands are elevated, and accordingly, recommendations are for increased weight gain in comparison to singleton pregnancies [23,24]. Not only how much weight was gained is critical, but also the timing of the gain is important to avoid an adverse outcome [25].

A study conducted by the same group as this study examined outcomes of twin pregnancy following a short IPI. The rationale of that study was that if the “nutrient depletion theory” is correct, it would be expected to see a greater effect in twin pregnancies following a short IPI in comparison to twin pregnancies following an optimal IPI [26]. However, in the multivariate analysis, no significant difference was found between the study groups. One possible reason for those findings, as mentioned in the original article, is that the high rate of iatrogenic PTB in twin gestations, especially in monochorionic diamniotic twins that participated in that study, masked the effect of short IPI on PTB.

In the current study, the outcomes of a singleton pregnancy following a short IPI were examined, and a higher rate of PTB was found in the short IPI group. This finding might reinforce once again the nutrient depletion theory. Following a twin pregnancy during which the maternal nutritional demand is increased, the body needs time to recover and refill its reservoirs. If these demands are not met, then the subsequent pregnancy begins with a deficit of essential nutrients. In these cases, in contrast to the previous study, no iatrogenic PTB is caused, and the adverse outcome of PTB can be better observed. It is also possible that the association that was found between short IPI and PTB is because both short IPI and PTB are more common in women of certain socioeconomic status. Women with short IPI are recognized as belonging to low socioeconomic status and tend to smoke, and they are less likely to be married and more likely to be younger than 20 years at the time of the second delivery. These are also risk factors for PTB [27]. Unfortunately, many socioeconomic factors were not available in this retrospective study, but there is a possibility that the interval itself is not the cause for the higher risk of PTB; rather, the common background characteristics are.

As mentioned, in the univariate analysis, an association was found between short IPI following a twin pregnancy and higher rates of placental abruption and retained placenta. The association between short IPI and placental abruption has been reported previously [28]. A possible explanation of this association could also be related to the previously mentioned theory of abnormal remodeling of the blood vessels in the endometrium among patients with short IPI [21].

When assessing spontaneous and induced PTB separately, Short IPI was associated only with induced PTB. Although the sample size of the study was not powered to examine these outcomes, this finding may support the theory of abnormal remodeling of the blood vessels in the endometrium as growth restriction and pre-eclampsia are common causes of induced PTB.

### 4.1. Clinical Implications

As IPI is a modifiable risk factor, informing women who deliver twins about the implications of conceiving shortly following the twins’ birth should be carried out in the postpartum visit. It is important to discuss with them the adverse outcomes associated with short IPI in pregnancy following multi-fetal gestation and inform them of the minimal recommended duration to optimize birth spacing. This should be balanced with maternal age, the need for fertility treatments, and future reproductive wishes. Such a discussion and recommendations cannot rely solely on this study, hence until more studies explore what we have found, we should use data regarding short IPI following a singleton delivery, and it seems reasonable to recommend an IPI of 18 months or at least 7 months, which is a similar recommendation to that of the American college of obstetrics and gynecology (ACOG) [14].

### 4.2. Research Implications

In the current study, increased risk of PTB, anemia (Hb < 11 gr%) on admission to labor, and placental abruption was found to be associated with short IPI in the unique population of women with pregnancy subsequent to multifetal gestation. We found no previous study that examined the association between IPI and adverse outcomes in the population of women with pregnancies following twin delivery. However, the correlation between short IPI and PTB, anemia, and placental abruption was found in studies that evaluate singleton pregnancies [20,28,29]. Nevertheless, further research is needed to strengthen those findings in populations from other countries and in order to explore alternative interventions that may decrease adverse outcomes among patients who conceived less than six months after the delivery of twins. In the current study, information regarding the etiology of the PTB was not available. This important information should be assessed in future studies to understand the causes of PTB better. Finally, the sample size of the different study groups is small, and larger cohorts are needed.

Our study had several strengths, notably the uniqueness of the study cohort. Our relatively large population with high parity enables us to assess this relatively uncommon subgroup of women with short IPI following a twin gestation in a developed country with advanced medical services. Furthermore, the study’s data were based on data retrieved from computerized medical records that are constantly updated in real-time at the point of care.

Our study also has several limitations. It is a retrospective study with inherent biases, and despite the large cohort, the relatively low number of women with short IPI resulted in a small sample size, which limited informative subgroup analyses. We are aware of the fact that our data are missing important information about patients’ ethnic background, socioeconomic status, or substance abuse, factors that are not included in our computerized medical database and may influence the rates of PTB. Furthermore, there may be other unknown confounding factors. In addition, our database includes only potentially viable deliveries as we excluded women who had pregnancy loss before the viability period in our country (22 weeks). Thus, this study did not assess the number of miscarriages following different IPIs, which is an important outcome. Although it is a multicenter center study, the population included in the study is relatively homogenous, which may impede the generalization of findings.

In conclusion, in a population who conceived following a twin pregnancy, short IPI is independently associated with PTB. Further studies are warranted to assess whether it is a true association or a surrogate for socioeconomic status.

## Figures and Tables

**Table 1:** Characteristics of women, pregnancies, and deliveries in the cohort.

	Optimal IPI (18–48 Months) *n* = 1142	Short IPI (0–6 Months) *n* = 160	*p* Value
Maternal age, years	32.1 ± 4.6	30 ± 5.3	<0.01
Previous miscarriages	301 (27.3%)	43 (29.9%)	0.52
Previous miscarriages ≥ 3	34 (3.1%)	8 (5.6%)	0.12
Gravidity	4.4 ± 2.5	4.4 ± 2.9	0.99
Parity	4 ± 2.2	3.9 ± 2.4	0.57
Interpregnancy pregnancy interval, months	29.9 ± 8.4	4.1 ± 1.2	<0.01
Smoking	21 (2%)	4 (3%)	0.45
Previous cesarean delivery in twin pregnancy	576 (51.3%)	52 (33.2%)	<0.01
Previous cesarean delivery, any	588 (51.5%)	54 (33.8%)	<0.01
Fertility Treatments	118 (10.7%)	2 (1.4%)	<0.01
Obesity (BMI ≥ 30)	52 (17.7%)	5 (17.2%)	0.95
Induction of labor	94 (8.9%)	19 (12.8%)	0.13
Trial of labor after cesarean	390 (35.4%)	33 (22.9%)	<0.01
Meconium-stained amniotic fluid	144 (13.1%)	20 (13.9%)	0.78
Epidural analgesia	543 (48%)	84 (54.5%)	0.13

Data are mean ± standard deviation, number (%) or median [IQR]; BMI Body Mass Index.

**Table 2:** Maternal outcomes of singleton delivery following short versus optimal IPI from a twin delivery.

	Optimal IPI (18–48 Months) *n* = 1142	Short IPI (0–6 Months) *n* = 160	*p* Value
Hypertensive disorders of pregnancy	14 (1.3%)	2 (1.4%)	0.91
Diabetes (pre-gestational + gestational)	62 (5.7%)	4 (3.1%)	0.20
Anemia (Hb < 11 gr%) on admission to labor	91 (11.4%)	20 (20%)	0.01
Placental abruption	23 (2.1%)	7 (5.3%)	0.03
Gestational age at delivery	39.2 ± 1.6	38.9 ± 1.9	0.05
Gestational age at delivery < 37 week	47 (4.3%)	14 (9.7%)	<0.01
Gestational age at delivery < 34 week	8 (0.7%)	3 (1.9%)	0.13
Gestational age at delivery < 32 week	3 (0.3%)	1 (0.6%)	0.44
Gestational age at delivery < 28 week	1 (0.1%)	0 (0%)	0.71
Spontaneous preterm birth (<37 weeks)	29 (2.6%)	7 (4.9%)	0.13
Indicated preterm birth (<7 weeks)	18 (1.6%)	7 (4.9%)	0.01
Unplanned cesarean	65 (5.9%)	8 (5.6%)	0.87
Elective cesarean	189 (17.2%)	21 (14.6%)	0.44
Postpartum hemorrhage	81 (7.4%)	5 (3.8%)	0.12
Blood products transfusion	15 (1.9%)	1 (1%)	0.53
Maternal ICU admissions	1 (0.1%)	0 (0%)	0.72
Placenta Accreta/Percreta	1 (0.1%)	0 (0%)	0.72
Prolonged hospital stays *	16 (2%)	3 (3%)	0.52
Composite adverse maternal outcome **	131 (11.5%)	22 (13.8%)	0.40

Data are mean ± standard deviation; number (%); ICU Intensive care unit. * maternal hospitalization length ≥ 7 days after cesarean or >5 days after vaginal delivery. ** defined as at least one of the following: Placental abruption, chorioamnionitis, puerperal fever, retained placenta/placental fragments, postpartum hemorrhage, blood products transfusion, maternal intensive care unit (ICU) admissions, and prolonged hospitalization (hospitalization length ≥ 7 days after a cesarean or >5 days after vaginal delivery).

**Table 3:** Neonatal outcomes.

	Optimal IPI (18–48 Months) *n* = 1142	Short IPI (0–6 Months) *n* = 160	*p* Value
Birthweight	3361.5 ± 490.5	3283.4 ± 509.7	0.06
Birthweight ≥ 4000 g	83 (7.5%)	6 (4.2%)	0.14
LGA	164 (14.9%)	20 (13.9%)	0.75
SGA	94 (8.5%)	10 (6.9%)	0.52
5-Minute Apgar score < 7	13 (1.1%)	1 (0.6%)	0.56
Perinatal death	34 (3.1%)	9 (6.3%)	0.05
NICU admission	1 (0.1%)	0 (0%)	0.72
Composite adverse neonatal outcome *	252 (22.1%)	33 (20.6%)	0.68

Data are mean ± standard deviation; number (%); LGA Large for gestational age; NICU Neonatal intensive care unit; SGA Small for gestational age; * Composite adverse neonatal outcome defined as at least one of the following: Perinatal death, SGA, 5-min Apgar score < 7, neonatal asphyxia, meconium aspiration, jaundice, congenital malformations, transient tachypnea of the newborn, brachial plexus injury, mechanical ventilation, convulsions, hypoglycemia, sepsis encephalopathy, and intracranial hemorrhage.

**Table 4:** Multivariate logistic regression analysis for the association between baseline characteristics and preterm delivery < 37 weeks (Adjusted Odds Ratio).

	*p*-Value	aOR	95% CI	
Previous preterm delivery(<37 weeks)	<0.01	3.02	1.55	5.88
Anemia (Hb < 11 gr%) on admission to labor	0.01	2.54	1.26	5.13
Short IPI (<6 months) following multifetal pregnancy	0.03	2.39	1.12	5.11
Previous cesarean delivery, any	0.17	3.12	0.62	15.65
Hypertensive disorders of pregnancy	0.21	2.21	0.63	7.73
Previous miscarriages ≥ 3	0.36	1.35	0.71	2.59

CI Confidence Interval, aOR adjusted odds ratio. IPI Interpregnancy Interval.

**Table 5:** Multivariate logistic regression analysis for the association between short IPI and composite adverse maternal and neonatal outcomes (Adjusted Odds Ratio).

	*p* Value	aOR	95%CI	
Composite Adverse Maternal Outcome *				
Previous cesarean delivery	<0.01	1.72	1.21	2.46
Short IPI	0.14	1.47	0.89	2.42
Gravidity	0.45	0.92	0.74	1.14
Parity	0.69	0.95	0.74	1.22
Fertility Treatments	0.94	1.02	0.58	1.79
Composite Adverse Neonatal Outcome **				
Diabetes (pre-gestational + gestational)	<0.01	8.64	4.95	15.08
Previous cesarean delivery	<0.01	1.99	1.41	2.80
Anemia (Hb < 11 gr%) on admission to labor	0.01	1.85	1.17	2.92
Maternal age, years	0.43	0.99	0.95	1.02
Short IPI	0.52	1.19	0.70	2.04
Hypertensive disorders of pregnancy	0.88	1.10	0.32	3.74

CI Confidence Interval, aOR adjusted odds ratio IPI Interpregnancy Interval, CD Cesarean Delivery. * defined as at least one of the following: Placental abruption, chorioamnionitis, puerperal fever, retained placenta/placental fragments, postpartum hemorrhage, blood products transfusion, maternal intensive care unit (ICU) admissions and prolonged hospitalization (hospitalization length ≥ 7 days after a cesarean or >5 days after vaginal delivery). ** defined as at least one of the following: Perinatal death, SGA, 5-min Apgar score < 7, neonatal asphyxia, meconium aspiration, jaundice, congenital malformations, transient tachypnea of the newborn, brachial plexus injury, mechanical ventilation, convulsions, hypoglycemia, sepsis encephalopathy, and intracranial hemorrhage.

## Data Availability

Not applicable.

## Supplementary Materials

The following supporting information can be downloaded at: https://www.mdpi.com/article/10.3390/jcm12072576/s1, Table S1: Baseline maternal, labor, and delivery characteristics of the different IPI groups as compared to women with optimal IPI (reference group); Table S2: Maternal outcomes of the different IPI groups as compared to women with optimal IPI (reference group); Table S3: Neonatal outcomes of the different IPI groups as compared to women with optimal IPI (reference group).
