# Supplementary material for: Short Interpregnancy Interval Following a Multifetal Pregnancy: Maternal and Neonatal Outcomes
Source: J Clin Med. 2023 Mar 29;12(7):2576. doi: 10.3390/jcm12072576 (PMC10094927; doi:10.3390/jcm12072576)
Supplement: Supplementary file 1 [file jcm-12-02576-s001.zip › jcm-2186521-supplementary.pdf]

Journal of Clinical Medicine

Table S1. Baseline maternal, labor and delivery characteristics of the different IPI groups as compared to women with optimal IPI (reference group).

	Optimal IPI -18-48 months (*n*=1142)	IPI 7-17 months (*n*=825)	*p* value	IPI >49 months (*n*=387)	*p* value
**Maternal age** [a]	32.1±4.6	29.9±5.2	<0.01	34.8±4.3	<0.01
**Previous miscarriages**	301 (27.3%)	239 (31.7%)	0.04	91 (23.7%)	0.17
**Previous miscarriages≥3**	34 (3.1%)	26 (3.4%)	0.67	7 (1.8%)	0.19
**Gravidity**	4.4±2.5	4.5±2.8	0.51	3.9±2	<0.01
**Parity**	4±2.2	4±2.5	0.87	3.6±1.7	<0.01
**Interpregnancy pregnancy interval** [b]	29.9±8.4	12.2±3.3	<0.01	66.7±18.4	<0.01
**Smoking**	21 (2%)	13 (1.8%)	0.79	14 (3.9%)	0.05
**Previous cesarean delivery**	588 (51.5%)	387 (46.9%)	0.05	216 (55.8%)	0.14
**Previous cesarean delivery in twin pregnancy**	576 (51.3%)	380 (46.7%)	0.19	208 (54.8%)	0.23
**Fertility Treatments**	118 (10.7%)	42 (5.6%)	<0.01	76 (19.8%)	<0.01
**Hypertensive disorders of pregnancy**	14 (1.3%)	8 (1.1%)	0.68	13 (3.4%)	0.01
**Diabetes** [c]	62 (5.7%)	24 (3.2%)	0.01	31 (8.2%)	0.08
**Obesity (BMI>30)**	52 (17.7%)	34 (17.8%)	0.99	35 (31.8%)	<0.01
**Trial of labor after cesarean**	390 (35.4%)	299 (39.6%)	0.06	50 (14.2%)	<0.01
**Induction of labor**	94 (8.9%)	72 (9.1%)	0.84	109 (28.4%)	0.01
**Meconium-stained amniotic fluid**	144 (13.1%)	140 (18.5%)	<0.01	53 (13.8%)	0.71
**Epidural analgesia**	543 (48%)	414 (51.3%)	0.15	186 (48.4%)	0.89

Data are mean± standard deviation; number (%); BMI Body Mass Index. a-years, b-months, c- pregestational + gestational.

Table S2. Maternal outcomes of the different IPI groups as compared to women with optimal IPI (reference group).

	Optimal IPI -18-48 months (*n*=1142)	IPI 7-17 months (*n*=825)	*p* value	IPI >49 months (*n*=387)	*p* value
**Gestational age at delivery**	39.2±1.6	39.4±1.6	0.02	38.8±2	<0.01
**Gestational age at delivery<37 week**	47 (4.3%)	31 (4.1%)	0.87	24 (6.3%)	0.12
**Gestational age at delivery<34week**	8 (0.7%)	6 (0.7%)	0.94	5 (1.3%)	0.27
**Gestational age at delivery<32week**	3 (0.3%)	4 (0.5%)	0.41	3 (0.8%)	0.16
**Gestational age at delivery<28week**	1 (0.1%)	1 (0.1%)	0.82	2 (0.5%)	0.10
Spontaneous preterm birth (<37 weeks)	29 (2.6%)	19 (2.5%)	0.87	9 (2.3%)	0.76
Indicated preterm birth (<37 weeks)	18 (1.6%)	12 (1.6%)	0.94	15 (3.9%)	0.01
**Prolonged hospital stays, mother**	16 (2%)	7 (1.3%)	0.30	6 (2.2%)	0.85
**Placenta Accreta/Percreta**	1 (0.1%)	1 (0.2%)	0.80	0 (0%)	0.56
**Maternal ICU admissions**	1 (0.1%)	0 (0%)	0.40	0 (0%)	0.56
**Postpartum hemorrhage**	81 (7.4%)	47 (6.3%)	0.37	36 (9.4%)	0.21
**Placental abruption**	23 (2.1%)	15 (2%)	0.90	7 (1.8%)	0.75
**Blood products transfusion**	15 (1.9%)	10 (1.8%)	0.92	3 (1.1%)	0.38
**In labor cesarean**	65 (5.9%)	48 (6.4%)	0.68	38 (9.9%)	0.01
**Elective cesarean**	189 (17.2%)	77 (10.2%)	<0.01	85 (22.1%)	0.03
**Anemia (Hb<11gr%) on admission to labor**	91 (11.4%)	87 (15.8%)	0.02	37 (13.6%)	0.36

Data are mean± standard deviation; number (%); IPI Interpregnancy interval; ICU Intensive care unit; N/A Non applicable.

Table S3. Neonatal outcomes of the different IPI groups as compared to women with optimal IPI (reference group).

	**Optimal IPI -18-48 months (*n*=1142)**	**IPI 7-17 months (*n*=825)**	***p* value**	**IPI >49 months (*n*=387)**	***p* value**
**Birthweight**	3361.5±490.5	3374.4±505.2	0.57	3241.2±519.7	<0.01
**Birthweight >4000 grams**	83 (7.5%)	64 (8.5%)	0.46	25 (6.5%)	0.50
**Large for gestational age**	164 (14.9%)	108 (14.3%)	0.75	47 (12.3%)	0.21
**Small for gestational age**	94 (8.5%)	69 (9.2%)	0.64	29 (7.6%)	0.56
**5-Minute Apgar score < 7**	13 (1.1%)	8 (1%)	0.72	9 (2.3%)	0.09
**Perinatal death**	2 (0.2%)	5 (0.6%)	0.11	1 (0.3%)	0.75
**NICU admission**	34 (3.1%)	18 (2.4%)	0.37	23 (6%)	0.01
**Composite adverse neonatal outcome**	252 (22.1%)	154 (18.7%)	0.07	98 (25.3%)	0.19

Data are mean± standard deviation; number (%); IPI Interpregnancy interval, NICU Neonatal intensive care unit, TTN transient tachypnea of the newborn; N/A Non applicable.
